# Heparan Sulfate Biosynthesis Enzyme, *Ext1*, Contributes to Outflow Tract Development of Mouse Heart via Modulation of FGF Signaling

**DOI:** 10.1371/journal.pone.0136518

**Published:** 2015-08-21

**Authors:** Rui Zhang, Peijuan Cao, Zhongzhou Yang, Zhenzhen Wang, Jiu-Lin Wu, Yan Chen, Yi Pan

**Affiliations:** 1 Key Laboratory of Nutrition and Metabolism, Institute for Nutritional Sciences, Shanghai Institutes for Biological Sciences, Chinese Academy of Sciences, University of the Chinese Academy of Sciences, Shanghai, China; 2 MOE Key Laboratory of Model Animal for Disease Study, Model Animal Research Center of Nanjing University, Nanjing, China; 3 Institute of Biomedical and Pharmaceutical Technology, Fuzhou University, Fuzhou, China; Heart Science Centre, Imperial College London, UNITED KINGDOM

## Abstract

Glycosaminoglycans are important regulators of multiple signaling pathways. As a major constituent of the heart extracellular matrix, glycosaminoglycans are implicated in cardiac morphogenesis through interactions with different signaling morphogens. *Ext1* is a glycosyltransferase responsible for heparan sulfate synthesis. Here, we evaluate the function of *Ext1* in heart development by analyzing *Ext1* hypomorphic mutant and conditional knockout mice. Outflow tract alignment is sensitive to the dosage of *Ext1*. Deletion of *Ext1* in the mesoderm induces a cardiac phenotype similar to that of a mutant with conditional deletion of UDP-glucose dehydrogenase, a key enzyme responsible for synthesis of all glycosaminoglycans. The outflow tract defect in conditional *Ext1* knockout(*Ext1* ^*f/f*^:*Mesp1Cre*) mice is attributable to the reduced contribution of second heart field and neural crest cells. *Ext1* deletion leads to downregulation of FGF signaling in the pharyngeal mesoderm. Exogenous FGF8 ameliorates the defects in the outflow tract and pharyngeal explants. In addition, *Ext1* expression in second heart field and neural crest cells is required for outflow tract remodeling. Our results collectively indicate that *Ext1* is crucial for outflow tract formation in distinct progenitor cells, and heparan sulfate modulates FGF signaling during early heart development.

## Introduction

The heart is the first organ to form and function in vertebrate embryos. Cardiogenesis is a tightly controlled process conserved in different species and requires proper interactions of distinct cardiac progenitor cells. Disruption of inductive signaling between progenitor cells causes cardiac malformation. Abnormalities in the arterial pole of the heart account for over 40% of congenital heart diseases (CHD) [1].Outflow tract (OFT) remodeling is critical for maturation of the arterial pole. The outflow tract is derived from two populations of precursors, specifically, second heart field (SHF) and neural crest cells. The SHF consists of splanchnic mesoderm cells of the pharyngeal region dorsal to the heart tube. Cells in the SHF add to the outflow tract and contribute to the outflow myocardium [2]. Neural crest cells (NCCs) migrate from the neuroectoderm of the dorsal neural tube. This cell population contributes to cushion formation and dictates correct septation and alignment of the heart [3].

Glycosaminoglycans (GAGs), the major components of the extracellular matrix (ECM), play critical roles in regulating transport and signaling of numerous growth factors during embryonic development. GAGs are long linear polysaccharide chains consisting of repeat disaccharide units, categorized into heparan sulfate (HS)/heparin, chondroitin sulfate (CS), dermatan sulfate (DS), and keratan sulfate (KS) based on their composition, sulfation, and epimerization patterns [4]. UDP-glucose dehydrogenase (UGDH) converts UDP-glucose to UDP-glucuronic acid, the common precursor of all GAGs. This monosaccharide is incorporated into the backbone of polysaccharide by the actions of distinct polymerization enzymes. Exostosin glycosyltransferases(Ext) exclusively catalyze heparan sulfate polymerization [5]. Disruption of genes that encode GAG biosynthesis enzymes can have profound effects on embryonic development. For instance, *lzme* embryos with *ugdh* deletion undergo gastrulation arrest along with defects in mesoderm and endoderm migration owing to disruption of FGF, but not Notch or Wnt signaling [6]. *Ext1* mutant embryos have been shown to die at gastrulation, displaying a phenotype similar to that of *ugdh* mutants [7].FGF signaling is specifically regulated by GAGs [8]. Another earlier *in vitro* study demonstrated that the FGF-FGFR complex preferentially binds to sulfated heparan sulfate [9].Genetic analyses further disclosed that loss of heparan sulfate reduces FGF signaling via effects on FGF-FGFR interaction or regulation of FGF diffusion during eye development [10, 11].

The heart field migrates ventrally and fuses anteriorly to form a heart tube composed of inner endocardium and outer myocardium. The extracellular matrix (ECM)-rich cardiac jelly lies between these two layers to support cardiac cushion morphogenesis and subsequent cardiovascular development. GAGs are the major constituents of ECM in the heart [12],with critical roles in morphogenesis. *Ugdh* is essential for cardiac valve formation in *Jelly* zebrafish [13]. Recently, *ugdh* was identified as a novel candidate target gene inpatients with AV septal defects [14].*Cspg2*, a chondroitin sulfate proteoglycan, is also implicated in segmentation of the heart tube and AV cushion formation [15, 16]. Hyaluronan facilitates cell invasion in the cardiac AV canal by activating ErbB2−ErbB3 receptors [17].Disruption of the interactions of heparan sulfate with EGF leads to enlarged cardiac valves with hyperproliferation of mesenchymal cells in the AV region [18]. Microinjection studies in chicken embryos suggest that heparan sulfate is involved in heart looping [19].Previous reports have implicated a role of heparan sulfate proteoglycan in cardiovascular development [20, 21]. Recently, we discovered that loss of N-deacetylase/N-sulfotransferase (NDST) in neural crest cells results in a phenotype similar to that of DiGeorge syndrome [22].

In the current study, experiments with hypomorphic *Ext1* and three Cre-mediated conditional *Ext1* knockout mutants have disclosed a role of heparan sulfate in OFT formation in distinct progenitor cell populations. Depletion of *Ext1* in the mesoderm led to reduced contribution of SHF and NCCs to the OFT and disrupted FGF signaling in the pharyngeal mesoderm and OFT. The defects of these mutants could be partially rescued by exogenous FGF8b. Moreover, *Ext1* expression in SHF and NCCs was required for proper OFT alignment. Based on the collective data, we propose that *Ext1* regulates OFT formation, and heparan sulfate is critical in modulating FGF signaling in mouse heart development.

## Materials and Methods

### Ethics statement

This study was carried out in strict accordance with the recommendations in the Guide for the Care and Use of Laboratory Animals of the Chinese Academy of Sciences. The protocol was approved by the Institutional Animal Care and Use Committee (IACUC) of the Institute for Nutritional Sciences, Shanghai Institutes for Biological Sciences, Chinese Academy of Sciences(Approval Number 2010-AN-8).

### Mouse models

*Ext* ^*Neo-f/+*^ and *Mef2C-Cre* mice were obtained from MMRRC (http://www.mmrrc.org). *Mesp1Cre* transgenic mice were kindly provided by Dr. Yumiko Saga. *Wnt1-Cre*, *Ugdh* ^*f/f*^ and *ROSAmT/mG* mice are described in previous reports [11, 23]. All mice were maintained on a mixed BL6/129sv background. For defining the embryonic stage, the noon of the day that a vaginal plug was found was recorded as 'embryonic day 0.5' (E0.5).In embryos younger than the 40-somite stage, the developmental stage was determined based on somite number.

### RNA *in situ* hybridization

Embryos were fixed in paraformaldehyde (PFA), embedded in optimal cutting temperature compound (OCT) and sectioned at 10μm, followed by *in situ* hybridization with digoxigenin-UTP–labeled riboprobe, as described previously [10]. The probes used in this study were as follows: *Ext1* (NM_010162, 820–3061), *ugdh* (NM_009466, 227–1708), *Pitx2* [24], *Wnt11*, *bmp4* and *mlc2v* [25], Sox9 [26], *Six1*, *Tbx1* and *Isl1* (kindly provided by B. Zhou), *Crabp1* (kindly provided by S.J. Conway), *Msx1* (kindly provided by R.E.Maxson Jr), *Mef2c* [27], *PEA3*, *Er81* and *Erm* [10].At least three embryos per genotype were analyzed for each probe.

### Histology, immunohistochemistry, and ink injection

Embryos were collected, fixed, embedded in paraffin blocks, and sectioned at 7μm. Slides were deparaffinized, rehydrated, and stained with H&E according to standard protocols. Immunohistochemical slides were initially treated for antigen retrieval. Briefly, slides were boiled in 10mM citrate buffer (pH6.0) for 30min before staining with antibodies specific for CD31 (clone 390, BD Pharmingen, USA), phospho-Erk (Cell Signaling Technology, USA), Ki67 (Abcam, USA), or 10E4 (Seikagaku, Tokyo, Japan). The TUNEL assay was performed on cryostat sections using an *in situ* cell death detection kit (Roche, Indianapolis, IN, USA).To examine aortic arch arteries, India ink was diluted 50% in PBS and injected into the left ventricle of the heart. Embryos were immediately fixed in PFA overnight, followed by a series of methanol/PBS to 100% methanol. Embryos were finally cleared in two volumes of benzyl benzoate and one volume of benzyl alcohol. At least three embryos per genotype were used for each assay.

### RNA isolation and real-time quantitative PCR

Embryos at different somite stages were collected. RNA was extracted with TRIzol reagent (Invitrogen, Carlsbad, CA, USA)and subsequently treated with RNase-free DNase I. Reverse transcription with oligo(dT) primer was performed using the SuperScript First-Strand Synthesis System(Invitrogen). Quantitative PCR analyses were performed with the SYBR Green PCR system, using actin as an internal control for normalization. Primers for each gene are listed as follows: 5’-GTCATCCATGCTGTGACTCC-3’ and 5’-GGCTTGTCACAATTCCACAG-3’ for *Ext1*, 5’-ACGTGTACAAGTTTGTGTGCGAGC-3’ and 5’-ATCCAAGTGGGACAAAGGGACTGT-3’ for *PEA3*, 5’-TCCAGAACCTGGATCACAGCAACA-3’ and 5’-GGCTTTCAGGCATCATCTTTGGCA-3’ for *Erm*, and 5’-GATCATTGCTCCTCCTGAGC-3’ and 5’-ACTCCTGCTTGCTGATCCAC-3’ for β-actin.

### Explant culture

OFT explants were cultured as described previously [28]. Briefly, OFT at the 12- to 14-somite stage was dissected, divided into halves in M199, and placed with endocardium facing down on the drained rat collagen gel saturated with culture medium (M199 supplemented with 1% FBS and insulin-transferrin-selenium). Explants were allowed to attach for 12 h, and medium added with or without FGF8b (100ng/μl, BD Biosciences, R&D Systems, USA). Explants were subsequently cultured for 24–48 h. After removal of OFT, pharyngeal explants connecting to the second pharyngeal arches were dissected and cultured as described previously [29]. Explants were placed on a collagen gel and cultured in M199 supplied with or without FGF8b. Cell migration was measured based on the greatest migrating distance to the explant edge after 24h culture, and assessed with Image J software.

### Statistical analysis

Statistical significance was analyzed with the online tool in GraphPad QuickCalcs (http://www.graphpad.com/). A paired two-tailed Student’s *t* test was used for analysis of cell proliferation and qRT-PCR. Data are presented as mean ± SEM. Phenotypic data were analyzed with Fisher’s exact test, with *P*< 0.05 considered as statistically significant.

## Results

### Proper expression of *Ext1* is required for cardiogenesis

**Fig 1:**
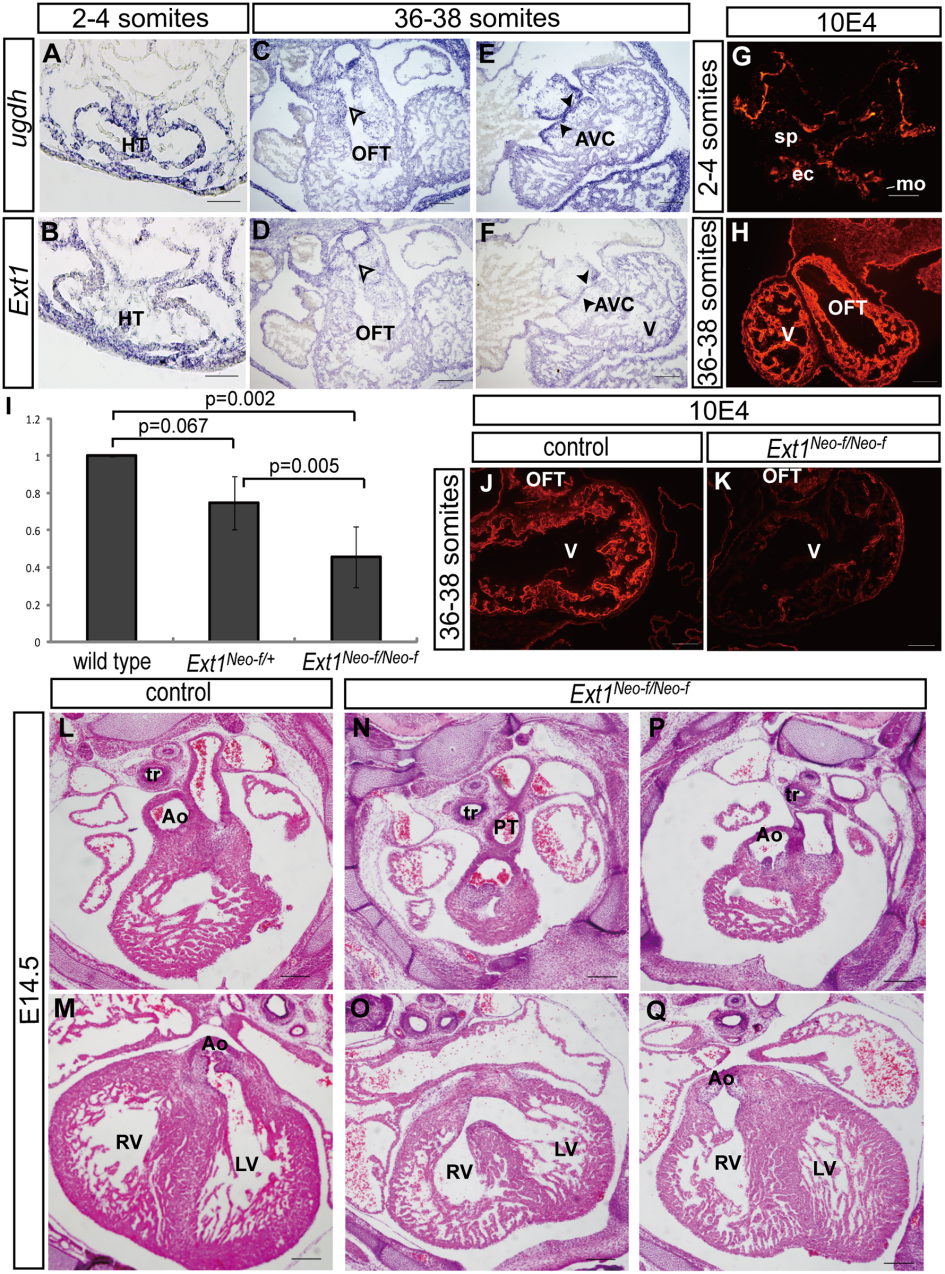
Heparan sulfate is required for heart development. (A–F) *In situ* hybridization was performed to examine *ugdh* and *Ext1*expression in embryos at the 2- to 4-somite stage(A,B) and 36- to 38-somite stage (C–F).Open arrowhead and closed arrowheads signify endocardium of the OFT cushion and AV cushion, respectively. (G,H)10E4 immunostaining was used as a parameter to detect *ugdh* and *Ext1*products at the 2- to 4-somite stage(G) and36- to 38-somite stage(H). (I) Determination of *Ext1* expression viaquantitative RT-PCR was performed with heart tissues of the wild-type, *Ext1* ^*Neo-f/+*^ and*Ext1* ^*Neo-f/Neo-f*^ embryos at the 25- to 26- somite stage. The *Ext1* mRNA level in wild-type heart was defined as 1. Four embryos were used for each genotype. (J,K) The Ext1 product was observed with 10E4 immunostaining in wild-type (J) and*Ext1* ^*Neo-f/Neo-f*^(K) embryos at the 36- to 38-somite stage. (L–Q)H&E analysis of the heart sections of control embryos (L,M) and*Ext1* ^*Neo-f/Neo-f*^ embryos (N–Q) at E14.5.Ao: aorta, ec: endocardial cells, LV: left ventricle, mo: myocardial cells, OFT: outflow tract; PT: pulmonary trunk (PT), RV: right ventricle, sp: splanchnic mesoderm, tr: tracheal, V: common ventricle.Scale bar: A,B: 50μm;C–H and J–Q: 100μm.

We initially examined the expression patterns of *ugdh* and *Ext1* in mouse embryos. Both genes were clearly expressed in the heart tube as early as at the 2- to 4-somite stage(Fig 1A and 1B).The *Ext1* transcript was detected in splanchnic mesoderm including the second heart field (Fig 1B). At the 36- to 38-somite stage, *ugdh* and *Ext1* mRNA were distributed throughout the heart, particularly endocardium of the OFT and AV cushions (Fig 1C–1F). Next, we used 10E4, a monoclonal antibody that recognizes N-acetylated and N-sulfated heparan sulfate, to identifythe*Ext1* and *ugdh* products. At the 2- to 4-somite stage, 10E4 staining was prominent in the splanchnic mesoderm(sp) and endocardium(Fig 1G). At the 36- to 38-somite stage, 10E4 signals were clearly observed in the OFT and common ventricular chamber(Fig 1H).These findings implicate a role of heparan sulfate in cardiac development.

Insertion of a neo-cassette is suggested to alter the expression of neighboring genes and cause unexpected phenotypes [30]. Here, we investigated conditional *Ext1* loxP mice containing a neomycin selection cassette flanked by FRT sites(named *Ext1* ^*Neo-f/Neo-f*^). Our data showed that only 25.6% of *Ext1* ^*Neo-f/Neo-f*^ mice survived after birth (78 pups) (Table 1). Analysis with qPCR revealed a 50% reduction in the *Ext1*transcriptin *Ext1* ^*Neo-f/Neo-f*^mouse hearts, compared with wild-type mice at the 25- to 26-somite stage (Fig 1I).*Ext1* ^*Neo-f/Neo-f*^ mice were therefore considered *Ext1* hypomorphic mutants. Although 10E4 could not detect all types of heparan sulfate, it was effective as a parameter for detecting *Ext1*. We consistently observed a decrease in 10E4 staining in OFT and the ventricle in *Ext1* ^*Neo-f/Neo-f*^ mice at the 36- to 38-somite stage (Fig 1K). Histological examination revealed ventricular septal defects in 80% *Ext1* hypomorphic embryos(VSD) at E14.5 (Table 1).About 67% of the hypomorphic mutants had cardiovascular defects, including persistent truncus arteriosus (PTA) and double outlet right ventricle (DORV) (Fig 1N–1Q). In addition, thin ventricular wall and non-compact ventricle were observed in 50% hypomorphic embryos (Fig 1O). Our results collectively suggest that cardiovascular development is sensitive to the *Ext1* dose.

**Table 1:** Summary of the cardiac phenotype in *Ext1* mutants.

Mutant	Cardiac phenotype	Stage of lethality
*Ext1* ^*Neo-f/Neo-f*^	~80% embryos with VSD, 67% embryos with PTA/DORV, 50% embryos with non-compact ventricle	E15.5 to birth, with 25.6%survival
*Ext1* ^*f/f*^:*Mesp1Cre*	Retarded growth, hypoplastic OFT and pharynx, distended heart tube, incomplete heart looping, thin common ventricle	E10.5 to E11.5
*ugdh* ^*f/f*^:*Mesp1Cre*	Retarded growth, hypoplastic OFT and pharynx, distended heart tube, incomplete heart looping, thin common ventricle	E10.5 to E11.5
*Ext1* ^*f/f*^:*Mef2cCre*	persistent truncus arteriosus	Not determined
*Ext1* ^*f/f*^:*Wnt1Cre*	persistent truncus arteriosus	At birth

### *Ext1* is indispensable for early cardiac development

**Fig 2:**
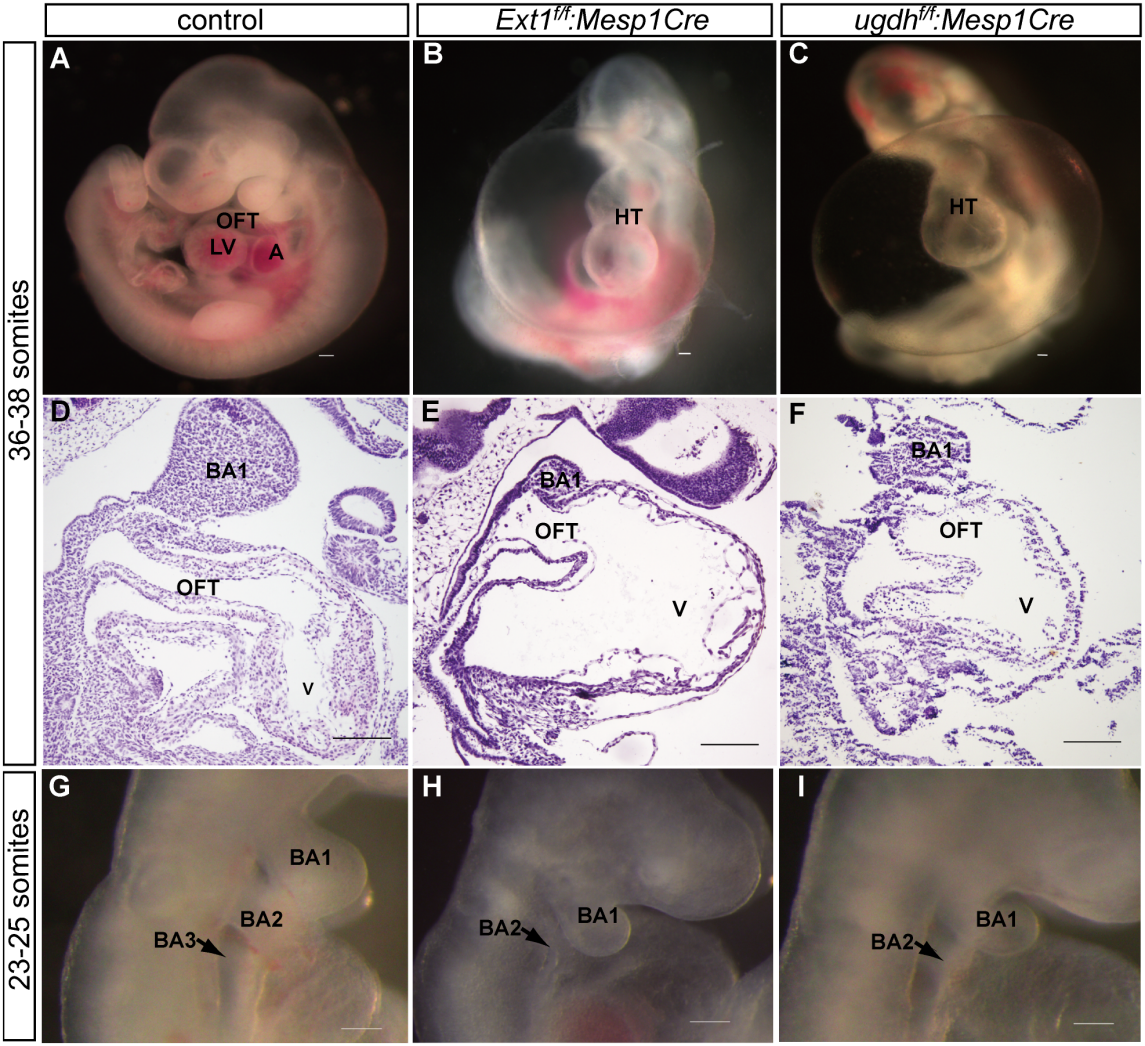
Conditional deletion of *Ext1*or *ugdh* in the mesoderm leads to severe cardiac defects. (A–C) Whole-mount view of control(A),*Ext1* ^*f/f*^:*Mesp1Cre*(B), and *ugdh* ^*f/f*^:*Mesp1Cre*mutant(C)embryos at the 36- to 38-somite stage. (D–F) H&E analysis of the heart section of control(D), *Ext1* ^*f/f*^:*Mesp1Cre*(E) and *ugdh* ^*f/f*^:*Mesp1Cre* mutant(F) embryos at the 36- to 38-somite stage. (G–I) Whole-mount view of branchial arches in control(G),*Ext1* ^*f/f*^:*Mesp1Cre*(H) and *ugdh* ^*f/f*^:*Mesp1Cre*(I) embryos at the 23- to 25-somite stage. A: atrium; BA: branchial arch, HT: heart tube, LV: left ventricle, OFT: outflow tract, V: common ventricle. Scale bar: 100μm.

To investigate whether *Ext1* is a direct effector of cardiovascular development, the hypomorphic allele was converted to an *Ext1* ^*f*^allele by crossing *Ext1* ^*Neo-f/Neo-f*^ with *Flp* recombinase transgenic mice. Conditional *Ext1* knockout in mesoderm was achieved by mating *Ext1* ^*f*^ with *Mesp1*:*Cre* transgenic mice, which were subsequently analyzed at E9.5, E10.5, and E11.5. No surviving *Ext1* ^*f/f*^:*Mesp1Cre* mutant mice were observed at E11.5, indicating death after the E10.5 stage. Compared to wild-type littermates, *Ext1* ^*f/f*^:*Mesp1Cre* mutants were obviously growth retarded and had massive pericardial effusion at E10.5 (Table 1, Fig 2).

At the 36- to 38-somite stage, outflow and inflow poles had converged in wild-type embryos (Fig 2A). However, *Ext1* ^*f/f*^:*Mesp1Cre* mutants exhibited a relatively straight and distended heart tube(Fig 2B), indicating blockage of heart looping and convergence. Histological analysis revealed a shortened outflow tract in the *Ext1* ^*f/f*^:*Mesp1Cre* mutants. In contrast to the narrow outflow lumen in wild-type embryos, the mutants displayed a broad OFT diameter (Fig 2D and 2E), suggesting hypoplasia of the outflow cushion in mutant embryos. Furthermore, at the 23- to 25-somite stage, wild-type embryos had well-formed first and second branchial arches and had begun to develop the third branchial arches (Fig 2G, arrow). In contrast, *Ext1* ^*f/f*^:*Mesp1Cre* mutants only displayed hypoplastic first and second branchial arches (Fig 2H, arrow).

As Ext1 is only responsible for heparan sulfate synthesis, we expected conditional deletion of *ugdh* in the mesoderm to induce a more severe phenotype than that of *Ext1* ^*f/f*^:*Mesp1Cre* mutants, since UGDH is critical for the production of multiple components of GAGs. However, *ugdh* ^*f/f*^:*Mesp1Cre* mutants displayed a similar phenotype to*Ext1* ^*f/f*^:*Mesp1Cre*mutants over different stages of development(Fig 2C, 2F and 2I). The *ugdh* ^*f/f*^:*Mesp1Cre* mutant embryos also exhibited poorly developed OFT and branchial arches, together with an incompletely formed cardiac loop, and died at the same stage as *Ext1* ^*f/f*^:*Mesp1Cre* mutants (Table 1). These findings support the essential requirement of mesodermal *Ext1* expression for heart morphogenesis. Furthermore, *Ext1* ^*f/f*^:*Mesp1Cre* mutants could recapitulate the cardiac phenotypes of *ugdh* ^*f/f*^:*Mesp1Cre* mutant mice.

### Development of OFT is affected by *Ext1* deletion

*NKX2*.*5* is a master gene for cardiogenesis. Deletion of *Ext1* did not alter *NKX2*.*5* expression at the 16- to 18-somite stage (S1A and S1B Fig). We further examined the genes expressed in cardiomyocytes (*hand1*, *mlc2a*, and *Myc*) and endocardium (*neuregulin*, CD31). Notably, these genes were not affected by deletion of *Ext1* at the 16- to 18-somite stage (S1C–S1L Fig), suggesting that specification of myocardium and endocardium is not altered in the*Ext1* ^*f/f*^:*Mesp1Cre* mutants.

**Fig 3:**
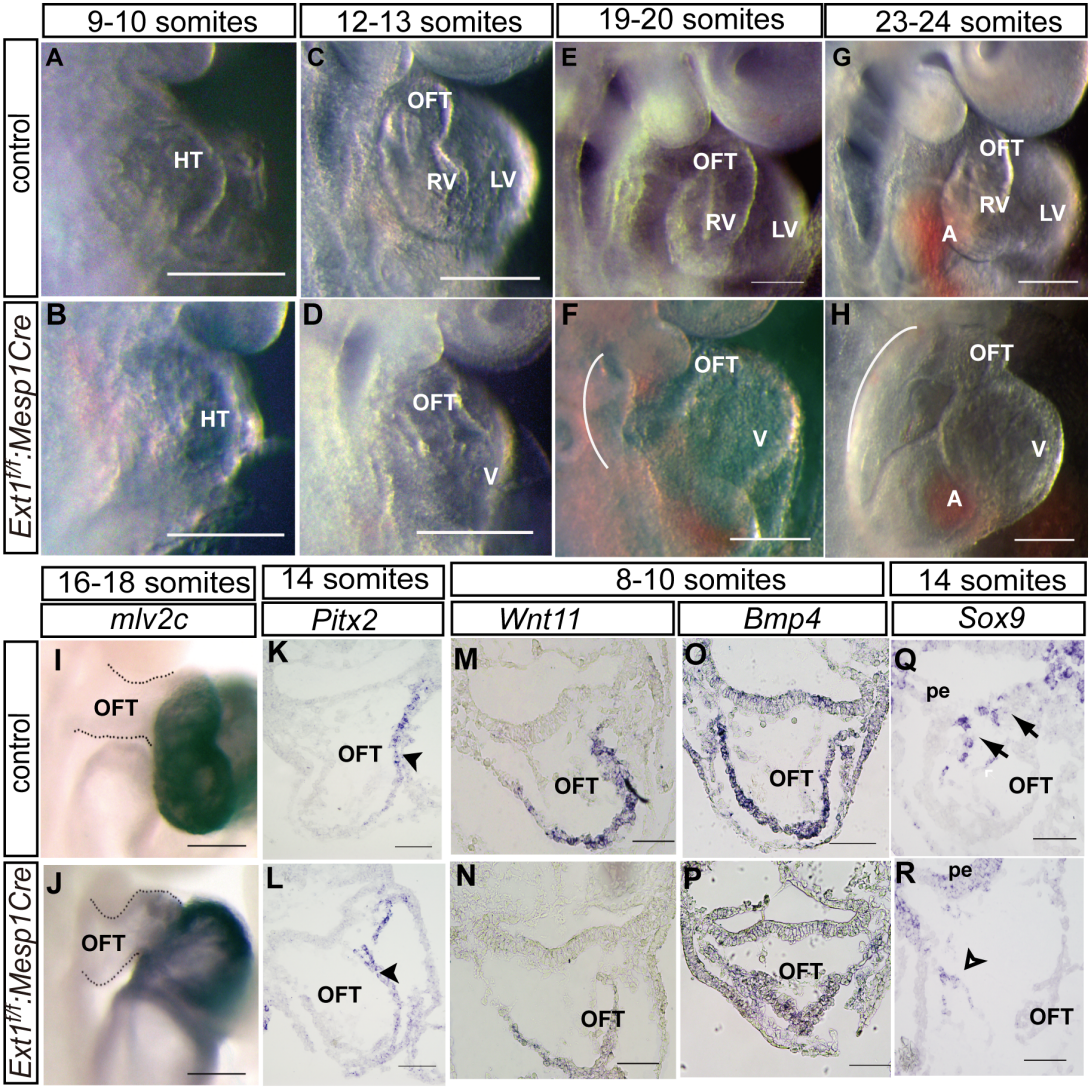
OFT defects in*Ext1* ^*f/f*^:*Mesp1Cre* mutant embryos. (A–H)Whole-mount view of the OFT phenotype in control and *Ext1* ^*f/f*^:*Mesp1Cre* embryos from the right lateral side at different stages of development. Arches indicate the underdeveloped pharyngeal region. (I,J)*mlc2v* expression was analyzed via whole-mount *in situ* hybridization. (K–R) *In situ* hybridization was used to examine the expression patterns of *Pitx2* at the 14-somite stage(K,L), *Wnt11*(M,N), *Bmp4* (O,P)at the 8- to 10-somite stage, and *Sox9* (Q,R) at the 14-somite stage. *Pitx2* expression remained unchanged in mutant embryos (K,L, arrowhead).Compared to control embryos (Q, arrow), the *Sox9* signal was reduced in the OFT cushion in mutant embryos (R, open arrow).HT: heart tube, LV: left ventricle, OFT: outflow tract, pe: pharyngeal endoderm,RV: right ventricle, V: ventricle. Scale bar: A–J, 100μm; K–R: 50μm.

We next investigated the gross morphology of *Ext1* ^*f/f*^:*Mesp1Cre* mutants at serial stages.*Ext1* deletion led to progressive growth failure in the OFT. At the 9- to 10-somite stage, the heart tube in mutant embryos was indistinguishable from control embryos (Fig 3A and 3B). At the 12- to 13-somite stage, both wild-type and *Ext1* ^*f/f*^:*Mesp1Cre* embryos had developed obvious cardiac asymmetry owing to heart looping (Fig 3C and 3D). At the 19- to 20-somite stage, the primary heart tube continued elongation and converged. The atrium was immediately posterior to the OFT and invisible from the right lateral view. Correspondingly, the outflow tract was lengthened and the right ventricle developed in the control embryos (Fig 3E).In contrast, in *Ext1* ^*f/f*^:*Mesp1Cre* mutant embryos, both elongation and looping of the heart tube were arrested (Fig 3F).The ventricle region bulged out and did not continue to turn rightwards in the mutants. A gap between the atria and OFT was observed, indicating blockage of the convergence process. The first branchial arches appeared smaller in the mutant than control embryos. These defects became more obvious at the 23- to 24-somite stage (Fig 3H). Furthermore, the right ventricle was not well formed in mutant embryos. At the 19- to 24-somite stage, pharyngeal region in the mutants was underdeveloped and much thinner than that in control embryos (Fig 3F and 3H, arch). As the OFT and right ventricle are derived from SHF cells, the reduced pharyngeal region is possibly responsible for hypoplasia of the OFT in mutant embryos.

The *mlc2v* marker was used to examine the ventricular myocardium and A/V junction [31].*In situ* hybridization revealed the presence ofm*lc2v* mRNA in the proximal outflow tract in *Ext1* ^*f/f*^:*Mesp1Cre* embryos at the 16- to 18-somite stage, suggesting that part of the mutant OFT has ventricular identity (Fig 3I and 3J). However, early heart looping was not affected by *Ext1* deletion. *Pitx2*, one of the genes involved in right–left side patterning, was expressed at similar levels in the*Ext1* ^*f/f*^:*Mesp1Cre* mutant and wild-type mice at the 14-somite stage(Fig 3K and 3L, arrowhead).

We further analyzed the key genes required for OFT formation. *Wnt11*, a myocardial marker of OFT, was barely detected in mutant heart at the 8- to 10-somite stage (Fig 3M and 3N). *Bmp4* is another marker for outflow myocardium [32]. Deletion of *Ext1* abolished *Bmp4* expression in the OFT at the 8- to 10- somite stage (Fig 3O and 3P).*Sox9* is activated when endocardial cells undergo epithelial–mesenchymal transformation (EMT) in the cushion region [33]. In contrast to strong distribution in the OFT cushion in control embryos (Fig 3Q), a weak *Sox9* signal was observed in the endocardium of the OFT cushion inmutants at the 14-somite stage (Fig 3R), suggesting that these endocardial cells are defective in EMT. Based on these findings, we propose that deletion of *Ext1* triggers defects in the OFT.

### Impaired contribution of the second heart field and neural crest cells to OFT upon *Ext1* deletion

**Fig 4:**
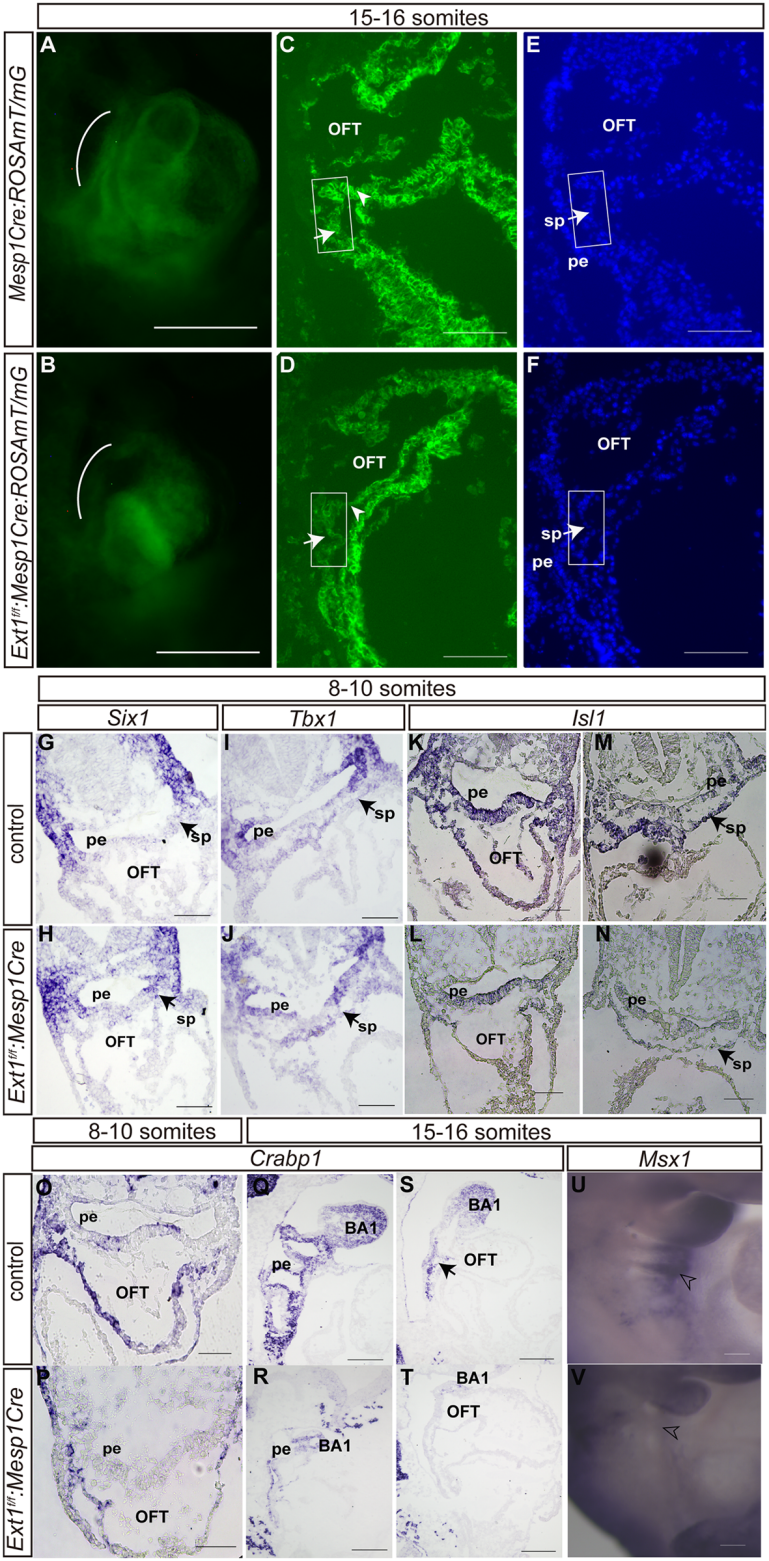
Both SHF and neural crest cells are affected in *Ext1* ^*f/f*^:*Mesp1Cre* mutant embryos. (A–F)Cell lineage analysis in *Mesp1Cre*and *Ext1* ^*f/f*^:*Mesp1Cre* mice at the 15- to 16-somite stage on whole-mount embryos(A,B) and sagittal cryosection(C,D). Sections(C,D) were counterstained with Hoechst(E,F). The pharyngeal region is indicated by the white arch. Cells in the boxed region were used for quantitation.(G-V) *In situ* hybridization was applied to analyze expression patterns of *Six1* (G,H), *Tbx1* (I,J), *Isl1* (K–N) at the 8- to 10-somite stage, *Crabp1*(O–T) at the 8- to 16-somite stage and *Msx1* (U,V) at the 15- to 16-somite stage. Arrowheads indicate OFT. Open arrowheads indicate the pharyngeal arch. Arrows signify the splanchnic mesoderm/SHF.BA: branchial arch, OFT: outflow tract, pe: pharyngeal endoderm, sp: splanchnic mesoderm. Scale bar: A–F, U, V: 100μm; G–T: 50μm.

Progenitor cells from the SHF contribute to the outflow tract in early cardiogenesis [34]. Previous studies suggest that *Mesp1* is activated in almost all cardiac precursors [35]. Accordingly, we analyzed Mesp1 lineage-traced cardiac cells by crossing *ROSAmT/mG* transgenic mice with *Mesp1Cre* mice and *Ext1* ^*f/f*^:*Mesp1Cre* mice, respectively, at the 15- to 16-somite stage. *Mesp1+* progenitor cells and their progeny were labeled with GFP. We observed a weak GFP signal in the pharyngeal mesoderm and distal OFT in the *Ext1* ^*f/f*^:*Mesp1Cre* mutants (Fig 4A–4D). Furthermore, SHF cells were reduced by ~30% in mutant embryos (S2A Fig).This finding suggests that *Ext1* deletion impairs mesoderm-derived cell populations.

As the second heart field is a subdomain of pharyngeal mesoderm contributing to the outflow tract, we further examined alterations in the markers that define SHF at the earlier stages. At the 8- to 10-somite stage, control and *Ext1* ^*f/f*^:*Mesp1Cre* embryos displayed similar cell numbers in the splanchnic mesoderm (S2B Fig). *Six1* is transiently expressed in progenitor cells in the SHF and acts as an upstream regulator of FGF8 signaling during cardiovascular development [28]. No visible differences in *Six1* expression were detected between the control and mutant embryos at the 8- to 10-somite stage (Fig 4G and 4H). *Tbx1*, another marker of the SHF, regulates multiple genes in the SHF and contributes to OFT morphogenesis [36]. Expression patterns of *Tbx1* were not altered in mutants at the 8- to 10-somite stage (Fig 4I and 4J). *Isl1* is the key marker for SHF, and its loss leads to absence of OFT and right ventricle [25]. Interestingly, we observed downregulation of *Isl1* in the OFT and SHF in mutant embryos at the 8- to 10-somite stage (Fig 4K–4N). Reduced *Isl1* expression was observed in SHF in the mutant at the 16- to 18-somite stage, but not the ventral spinal cord (S2C–S2F Fig).These results clearly suggest that *Ext1* deletion affects cells in the SHF.

Neural crest cells represent another cell population that contributes to OFT formation. Neural crest cells are located around the pharyngeal arches in close apposition to the SHF. To address whether these cells are affected by *Ext1* deletion, we examined *Crabp1* expression. *Crabp1*was markedly reduced in the pharyngeal region in mutant embryos at different somite stages (Fig 4O–4T). The first branchial arch in the *Ext1* ^*f/f*^:*Mesp1Cre* mutants contained fewer*Crabp1*-positive cells than the corresponding controls at the 15- to 16-somite stage. *Msx1*, a gene regulating appropriate migration and differentiation of the neural crest-derived arch mesenchyme, was markedly downregulated in the pharyngeal region at the 15- to 16-somite stage (Fig 4U and 4V).Deletion of *Ext1* in the mesoderm caused reduction of neural crest cells in the pharyngeal apparatus. Accordingly, we suggest that OFT defects in the *Ext1* ^*f/f*^:*Mesp1Cre* mutant are attributed to dysregulation of both SHF progenitor and neural crest cells.

### FGF signaling in SHF and OFT is alteredupon*Ext1* deletion

**Fig 5:**
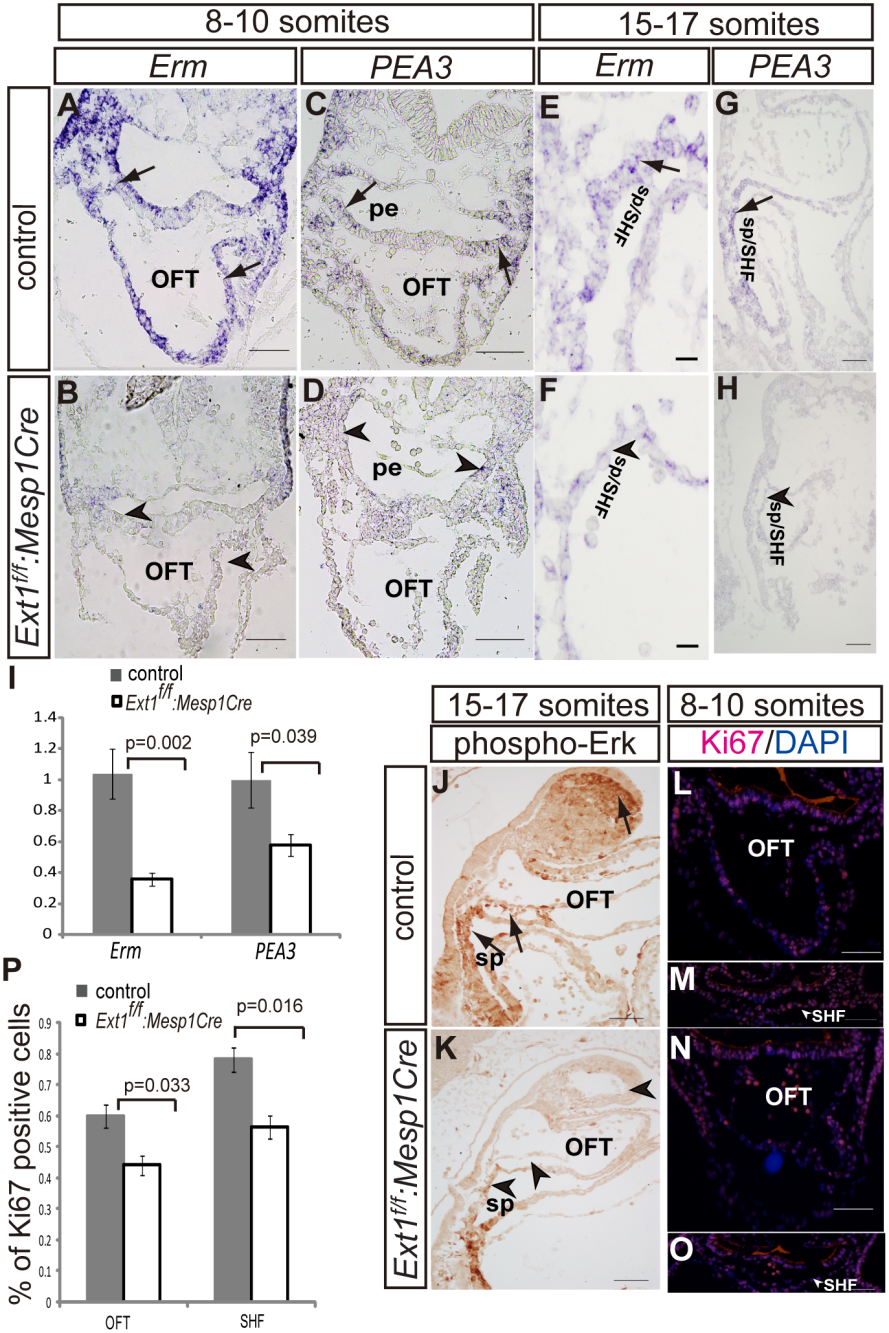
Deletion of *Ext1*in the mesoderm leads to reduced FGF signaling. (A–H) *In situ* hybridization was used to detect expression of *Erm*(A,B,E,F)and *PEA*3(C,D,G,H) in control and *Ext1* ^*f/f*^:*Mesp1Cre* embryos at different stages of development. (I) *Erm* and *PEA*3 mRNA levels were quantitated via qPCR at the 10-somite stage. *Erm* or *PEA3* transcript in control embryos was defined as 1. Four embryos were used for each allele. (J,K)Phosphor-Erk was identified by immunostaining at the 15- to 17-somite stage. The arrow indicates the signal detected in control, but not mutant embryos (arrowhead). (L–O) Cell proliferation was detected via Ki67 immunostainingin the outflow tract (L, N) and the second heart field (M, O) at the 8- to 10-somite stage. (P) Cell proliferation in the myocardium of OFT and SHF was quantitated, respectively, as the ratio of Ki67-positiveversus Hoechst-positive cells. Three embryos were used for each allele, and three sections counted for each embryo.OFT: outflow tract; pe: pharyngeal endoderm,sp/SHF: splanchnic mesoderm/second heart field. Scale bar: A–D, L–O: 50μm; G, H, J, K: 100μm, E, F: 10μm.

The FGF pathway is essential for the development of SHF and neural crest-derived structures. To determine whether FGF signaling is altered by *Ext1* depletion, we examined downstream genes of the FGF pathway. Although *FGF8* expression was not changed in the pharynx in *Ext1* ^*f/f*^:*Mesp1Cre* mutants at the 8–10 somite stage (S3A–S3D Fig), two downstream genes, *Erm* and *PEA*, were markedly downregulated in the pharyngeal region and splanchnic mesoderm upon *Ext1* deletion at this stage (Fig 5A–5D and 5I). Downregulation of *PEA3* and *Erm* was evident in the mutant at the 15- to 17-somite stage (Fig 5E–5H). The *Erm* mRNA level was reduced by about 60%, and the *PEA3* transcript decreased by 40% in the mutants at the 10-somite stage (Fig 5I). Consequently, an intracellular target of FGF signaling, phospho-Erk, was significantly decreased in SHF at the 15- to 17-somite stage (Fig 5J and 5K), especially in the anterior SHF where the cells are added to OFT. The phospho-Erk positive signal was observed in the distal cushion of the OFT in control embryos (Fig 5J, arrow), but not mutant embryos (Fig 5K). Furthermore, in *Ext1* ^*f/f*^:*Mesp1Cre* mutants, phospho-Erk was absent in the apical portion of the first branchial arches(Fig 5K) where neural crest-derived cells are mainly located.

As FGF signaling controls cell proliferation of cardiac progenitors, we used the general cell cycle marker, Ki67, to evaluate changes in cell proliferation. Our results showed a ~25% reduction inKi67-labeled cells in SHF and OFT at the 8- to 10-somite stages (Fig 5L–5P). Accordingly, we propose that *Ext1*deletion leads to disruption of FGF signaling in the pharyngeal mesoderm, subsequently affecting cell proliferation.

### FGF8 rescues the defects of OFT and pharyngeal explants induced upon *Ext1* deletion

**Fig 6:**
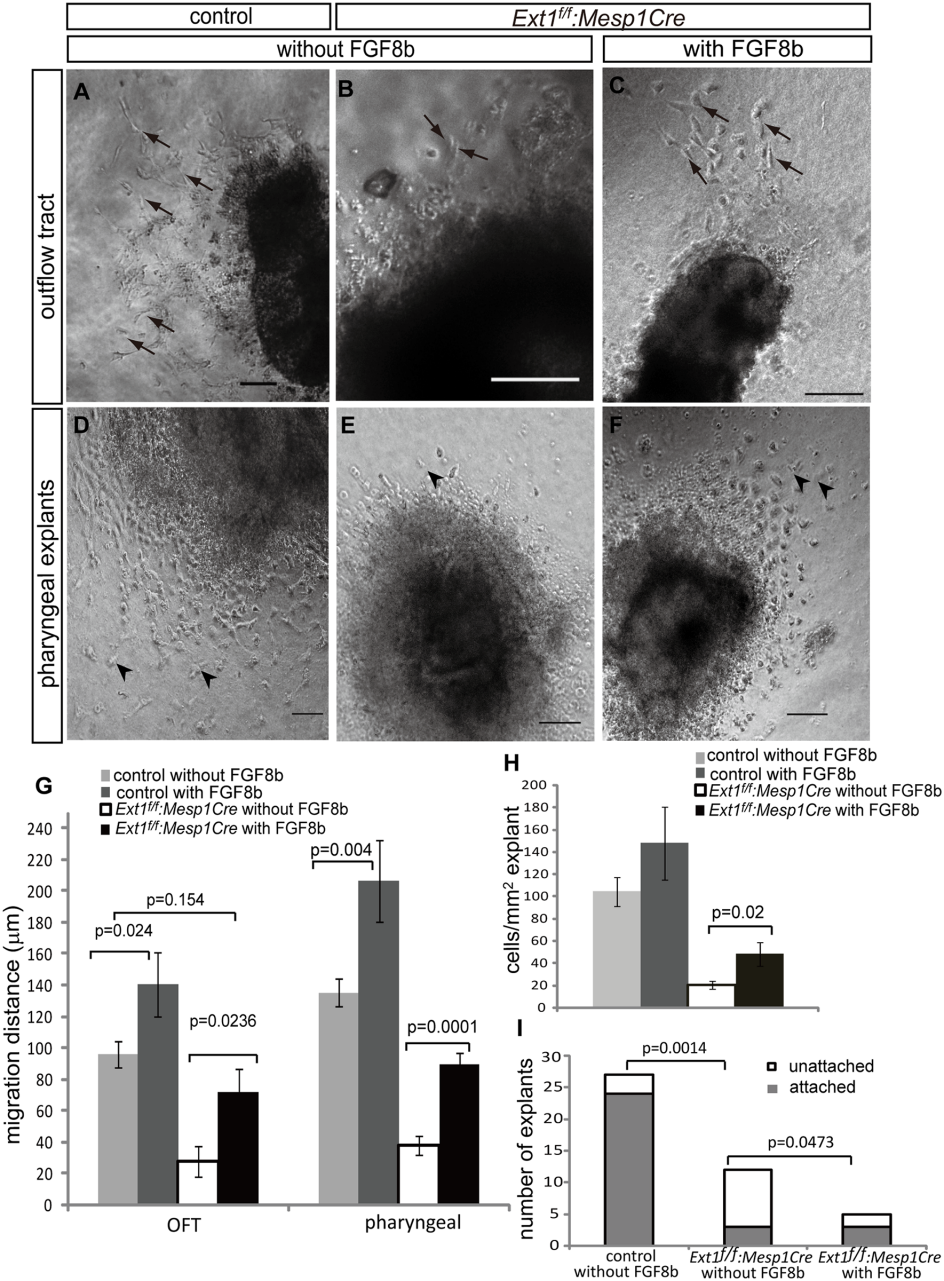
Defects in*Ext1* ^*f/f*^:*Mesp1Cre* embryos are ameliorated by exogenous FGF8b. (A–C) OFT explants of control embryos were cultured without exogenous FGF8b(A),and OFT explants of *Ext1* ^*f/f*^:*Mesp1Cre* embryos cultured without FGF8b(B) or with FGF8b(C). The arrow indicates cells undergoing EMT. (D–F) Pharyngeal explants of control embryos were cultured without FGF8b(D), and pharyngeal explants of *Ext1* ^*f/f*^:*Mesp1Cre* embryos cultured without(E) or with FGF8b(F). The arrowhead indicates migrated mesenchymal cells. (G,H) Quantitation of cell migration by evaluating the greatest distance to explants(G) and number of OFT cells invading the gel(H). We measured 24 control and 9 mutant explants cultured without FGF8b, and 10 mutant explants cultured with FGF8b. In total, 12 control explants cultured with FGF8b were used as the positive control. (I) Adhesion ability of OFT explants of the mutants was assessed after 24 h of culture. A–F: 50μm.

FGF8 is a major FGF ligand for SHF deployment, OFT extension and EMT. We observed a hypoplastic OFT cushion (Fig 2E) and altered EMT markers in the OFT cushion in *Ext1* ^*f/f*^:*Mesp1Cre* mutants(Fig 3Q and 3R). Next, we used a well-established explant system to examine whether exogenous FGF8 ligand can rescue dysregulated cell behavior in the OFT of mutant embryos [28]. In the explant system, cells undergo EMT, migration and invasion. EMT in the OFT was assessed by counting the mesenchymal cells that invaded the collagen gel and measuring the migration distance of cells after invasion. All cells invading the gel were derived from the mesoderm (S4A Fig). Mutant explants displayed a dramatic reduction in the number of mesenchymal cells invading the gel, together with a significantly shorter distance of invasion (Fig 6B, arrow, Fig 6G and 6H). In addition, OFT explants of the mutant embryos exhibited poor adhesion in the collagen matrix. Less than 50% of the explants of the *Ext1* ^*f/f*^:*Mesp1Cre* mutants were seeded onto the collagen gel (Fig 6I). However, upon addition of exogenous FGF8b, ~80% of the OFT explants of mutant embryos were able to attach onto the collagen gel, equivalent to that observed with control explants (Fig 6I). Furthermore, FGF8b induced a 2.6-fold increase in mesenchymal cell migration (Fig 6C and 6G). Although the number of mesenchymal cells was still lower than that in control embryos, FGF8b treatment enhanced the cell number two-fold in the *Ext1* ^*f/f*^:*Mesp1Cre* mutant (Fig 6H).

The pharyngeal apparatus consists of several cell types from the different germ layers, including the neural crest, pharyngeal endoderm, ectoderm, and mesoderm [37]. In the pharyngeal region, the neural crest-derived mesenchymal cells and pharyngeal mesoderm-derived SHF cells are critical for OFT morphogenesis. Migrating cells of pharyngeal explants included mesoderm-derived and nonmesoderm-derived cells (S4B Fig). Some mesenchyme-like cells migrated from the explants (Fig 6D), but this movement was reduced in pharyngeal explants of the mutant embryos (Fig 6E), indicative of a defect in migration upon *Ext1* deletion. Notably, administration of exogenous FGF8b improved cell migration of pharyngeal explants of the mutants (Fig 6F and 6G). Explants of control embryos were also cultured with exogenous FGF8b as a positive control. Following FGF8b stimulation, the OFT and pharyngeal explants displayed increased migration, but to a lesser extent than that observed in mutant embryos (Fig 6G). Based on these findings, we conclude that the defects caused by *Ext1* depletion can be partially rescued by exogenous FGF8.

### *Ext1* is required in both SHF and neural crest for OFT alignment

**Fig 7:**
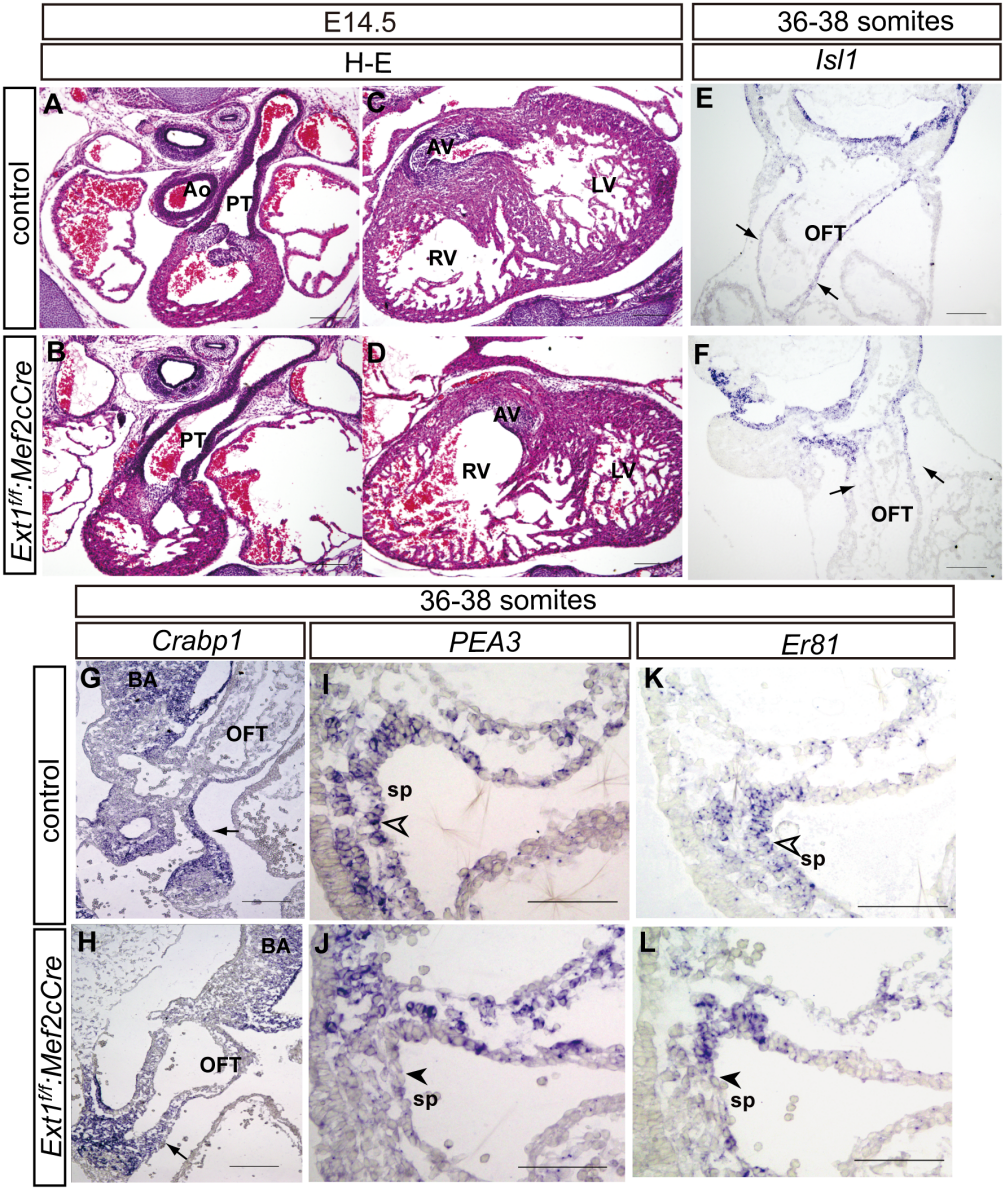
*Ext1* in SHF is required for OFT development. (A–D) H&E staining of heart sections of control embryos(A,C) and *Ext1* ^*f/f*^:*Mef2cCre* embryos(B,D) at E14.5. (E–L) Expression patterns of *Isl1*(E,F), *Crabp1*(G,H), *PEA3*(I,J) and *Er81*(K,L) were examined via *in situ* hybridization at the 36- to 38-somite stage. Restricted *Isl1* distribution in the OFT is marked by arrows(E, F). Expression of *Crabp1*in the mutant was not altered(G,H, arrow). Open arrowheads indicate *PEA3-* and *Er81-*positive signals in SHF cells of control embryos. These signals were reduced in*Ext1* ^*f/f*^:*Mef2cCre* embryos(J,L, arrowheads).Ao: aorta, AV: aorta valve, LV: left ventricle, OFT: outflow tract; PT: pulmonary trunk (PT), RV: right ventricle, sp: splanchnic mesoderm. Scale bar: A–H: 100μm; I–L: 50μm.

Since both the SHF and neural crest cells were affected by *Ext1* deletion in the mesoderm, we further examined whether *Ext1* is directly required by these cell populations during OFT formation. *Ext1* ^*f*^ and *Mef2cCre* transgenic mice were crossed to obtain a mouse line with *Ext1* deficiency in the SHF progenitors (*Ext1* ^*f/f*^:*Mef2cCre*). We observed a single outflow vessel in all the mutant embryos at E14.5 and E15.5 (5 of 5 mutants) (Fig 7B and 7D), suggesting failure in septation between aorta and pulmonary artery (Table 1).The SHF marker, *Isl1*, displayed no distinguishable differences in splanchnic mesoderm/SHF between control and mutant mice at the 36- to 38-somite stage, while *Isl1*-positive signals were less expanded into the outflow tract in mutant embryos (Fig 7E and 7F).No changes in *Crabp1* were detected in *Ext1* ^*f/f*^:*Mef2cCre* mutants, suggesting that neural crest cells are not affected in mutant embryos (Fig 7G and 7H, arrow). However, the levels of two FGF downstream genes, *PEA3* and *Er81*, were reduced in the SHFof*Ext1* ^*f/f*^:*Mef2cCr e*mutants (Fig 7I–7L), indicating a decrease in FGF signaling output in this region. Thus, loss of *Ext1* in the SHF appears to directly lead to OFT defects, accompanied by a reduction of FGF signaling.

**Fig 8:**
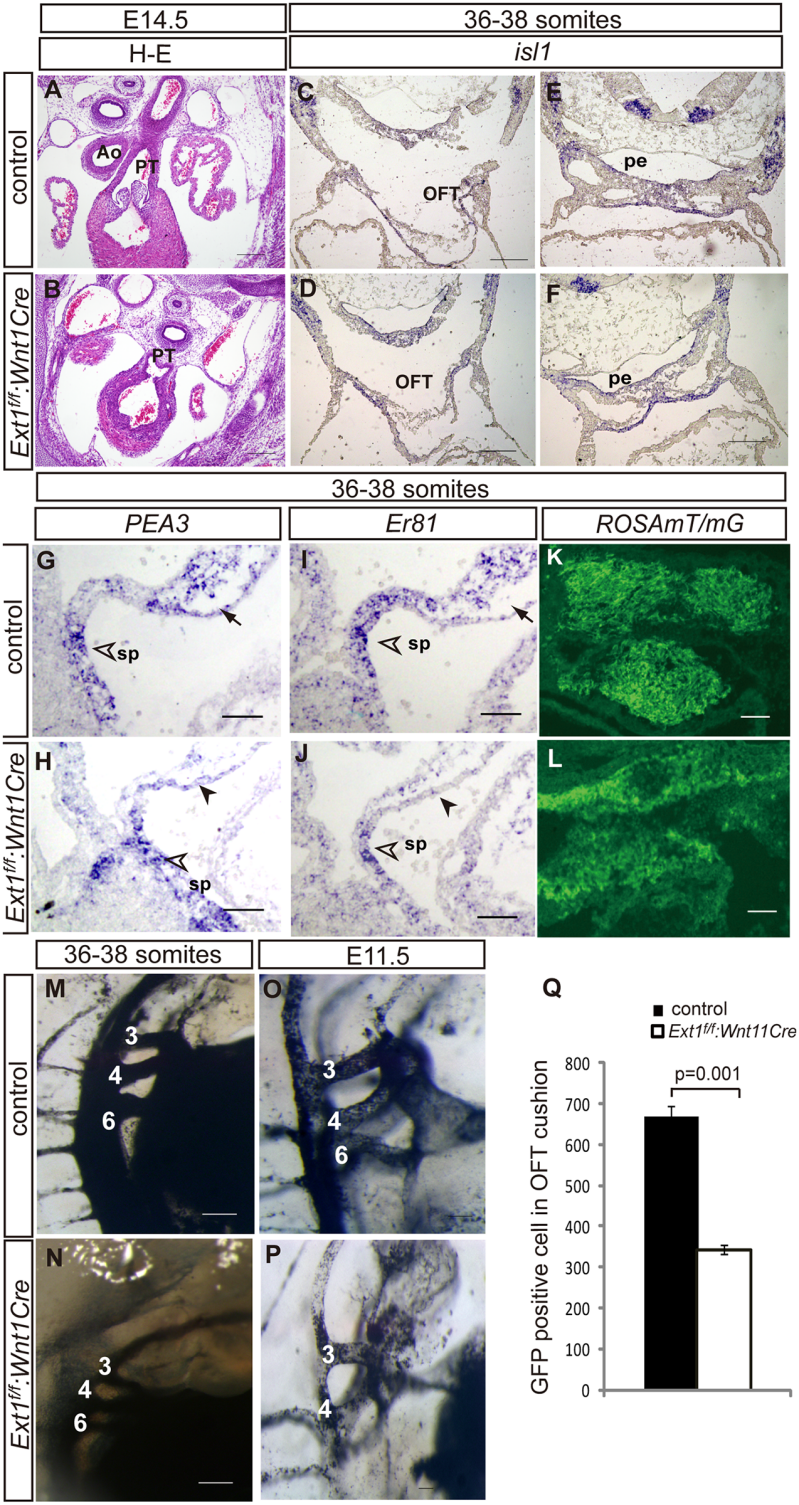
*Ext1* in neural crest cells is required for OFT development. (A, B) H&E staining in the heart section of control(A) and*Ext1* ^*f/f*^:*Wnt1Cre* embryos (B) at E14.5. (C–F) *Isl1* was expressed at similar levels in control and mutant embryos. (G–J) Expression patterns of *PEA3*(G,H) and *Er81*(I,J) were examined via *in situ* hybridization at the 36- to 38-somite stage. *PEA3* and *Er81* expression remained unchanged in the second heart field (open arrowheads), but was reduced in the OFT cushion of mutant embryos (arrows in G,I and arrowheads in H,J). (K,L)By crossing with *ROSAmT/mG*mice, neural crest-derived cells were labeled with GFP. Fewer GFP-positive cells are located in the OFT cushion at the 36- to 38-somite stage. (M-P) Aortic arch arteries were marked with India ink. The third, fourth and sixth aortic arch arteries were all labeled in control and mutant embryos at the 36- to 38-somite stage, but one of the sixth aortic arch arteries was absent in mutant embryos at E11.5. (Q) Quantitative results of neural crest-derived cells in the OFT cushion in K, L (three embryos were used for each allele, and three sections counted for each embryo). Ao: aorta, PT: pulmonary trunk, pe: pharyngeal endoderm;sp: splanchnic mesoderm. Scale bar: A–F, M–P: 100μm; G–L: 50μm.

*Ext1* conditional knockout in neural crest cells was achieved by crossing *Ext1* ^*f*^ with *Wnt1Cre* transgenic mice. All *Ext1* ^*f/f*^:*Wnt1Cre* embryos displayed an OFT defect, specifically, persistent truncus arteriosus at E14.5 (4 of 4 embryos) (Fig 8B, Table 1). Expression of *Isl1* remained unaltered in the OFT and SHF in *Ext1* ^*f/f*^:*Wnt1Cre* mice (Fig 8C–8F). Similar levels of*PEA3* and *ER81* in the second heart field were detected in both *Ext1* ^*f/f*^:*Wnt1Cre* mutant and control embryos (Fig 8G–8J). Signals of *PEA3* and *ER81* in the distal OFT cushion were reduced in *Ext1* ^*f/f*^:*Wnt1Cre* mutants (Fig 8H and 8J, arrowheads), suggesting that the FGF downstream signal is impaired in the OFT mesenchyme when *Ext1*is abolished in neural crest cells. Tracing of neural crest-derived cells using a cell lineage assay further revealed a~50% reduction in GFP-positive cells in the OFT cushion in *Ext1* ^*f/f*^:*Wnt1Cre* mutants, compared to controls, at the 36- to 38-somite stage (Fig 8K, 8L and 8Q). Using ink injection, aortic arch arteries were labeled in the embryos. The third, fourth and sixth aortic arch arteries were all marked in control and mutant embryos at the 36- to 38-somite stage. However, at E11.5, one of the sixth aortic arch arteries was missing in the one side of the mutant embryos (2 of 3 mutants) (Fig 8M–8P).Our data indicate that *Ext1* regulates the FGF signaling pathway in both SHF and neural crest cells.OFT defects in *Ext1* ^*f/f*^:*Mef2cCr*e and *Ext1* ^*f/f*^:*Wnt1Cre* mice may be caused by different progenitor cells.

## Discussion

In the present study, we investigated the role of *Ext1* in cardiovascular development using a variety of mouse models. ECM macromolecules, including proteoglycans and glycosaminoglycans, have been shown to play critical roles in heart development in different animal models. Hyaluronan and chondroitin sulfate are two abundant glycosaminoglycans in cardiac jelly. Ablation of hyaluronan synthase,*has2*, or chondroitin sulfate proteoglycan 2 (*Cspg2*)leads to aberrant cell migration in the cardiac cushion [16, 38]. Additionally, *has-/-* and *Cspg2-/-* mice display similar defects in heart development, including absence of right ventricle and short OFT [15]. The interactions between hyaluronan and CSPG2 may underlie the phenotype similarities [39]. HS, another type of glycosaminoglycan, regulates valve formation and cardiovascular development [18, 22]. In our experiments, specific disruption of the HS polymerase, *Ext1*, in the mesoderm, resulted in phenotypes similar to previously reported heart deformities in *has-/-*and *Cspg2-/-* mice, indicating overlapping functions among the individual components of GAGs in regulating cardiogenesis. Deletion of *ugdh* has been shown to result in loss of hyaluronan, chondroitin sulfate and HS. Interestingly, *Ext1* ^*f/f*^:*Mesp1Cre* and *ugdh* ^*f/f*^:*Mesp1Cre* mice displayed similar cardiac phenotypes. Our findings not only confirm the significance of glycosaminoglycans in cardiogenesis, but also support a critical role of HS in cardiac development, as HS loss only can recapitulate the cardiac phenotype induced by deletion of all GAGs in the mesoderm.

Gradual addition of SHF cells to the distal outflow tract facilitates elongation and remodeling of the heart tube. This process is mediated by multiple signal pathways from adjacent cells, including those from the pharyngeal endoderm and neural crest cells. Molecular exchange between the SHF and neural crest cells is an intricate mechanism essential for OFT morphogenesis. Ablation of cardiac neural crest cell leads to elevated FGF8 signaling, leading to overproliferation of SHF cells and failure of cell migration to the OFT from SHF [40, 41]. Loss of *Tbx3* in neural crest cells induces an increase in SHF proliferation via upregulation of FGF signaling [42].We used Cre driven by the *Mesp1* promoter to specifically delete *Ext1* in the mesoderm, but not the neural crest [43]. *Ext1* ^*f/f*^:*Mesp1Cre* mice had an underdeveloped pharyngeal region and reduced neural crest cells in the pharyngeal arches. Lack of *Crabp1*-positive cells near the distal outflow tract was additionally detected in mutant mice. This decrease in the neural crest cell number may thus contribute to hypoplastic OFT in mutant embryos.

SHF cells represent a population of Mesp1+ cells and belong to a subdomain of the pharyngeal mesoderm. We observed a thin Mesp1+ cell layer and reduced *Isl1*-positive cells in the splanchnic mesoderm of *Ext1* ^*f/f*^:*Mesp1Cre* mutants (Fig 4D and 4K–4N, S2 Fig). It appears that deletion of *Ext1* impairs the SHF cell population, with subsequent effects on SHF-derived structures. Our results raise the possibility that OFT defects are a secondary effect due to dysplasia of the pharyngeal mesoderm in *Ext1* ^*f/f*^:*Mesp1Cre* mutants. Defective SHF may arise as a result of reduced cell proliferation or impaired generation of precursors. Distinct from *Ext1* ^*f/f*^:*Mesp1Cre* mutant mice, ablation of *Ext1* in the SHF did not affect the SHF population in the pharyngeal region in *Ext1* ^*f/f*^:*Mef2cCre* mutants (Fig 7F). Furthermore, in contrast to *Ext1* ^*f/f*^:*Mesp1Cre* mutants, we did not observe the defect in the right ventricle in *Ext1* ^*f/f*^:*Mef2cCre* embryos (Figs 3H and 7D). Deletion of *Ext1* in the SHF did not trigger neural crest cell defects in the *Ext1* ^*f/f*^:*Mef2cCre* mutant as for *Ext1* ^*f/f*^: *Mesp1Cre* embryos. Considering that *Mesp1Cre* is activated earlier and to a wider extent than *Mef2cCre* in the cardiac progenitors in heart development, the severe defects observed in *Ext1* ^*f/f*^:*Mesp1Cre* mutants are possibly due to early disruption of *Ext1* in the mesoderm.

OFT morphogenesis requires the appropriate FGF signal levels [44–46].HS has been identified as a co-receptor of the FGF/FGFR complex that facilitates signal transduction [8] as well as a regulator in FGF ligand diffusion [11].Our study revealed that although *FGF8* mRNA expression remains unchanged, FGF downstream response genes are downregulated in the *Ext1* ^*f/f*^:*Mesp1Cre* embryos. Deletion of *Ext1* may suppress FGF signaling in the pharyngeal mesoderm, inhibiting cell proliferation of SHF and OFT. In the *Ext1* ^*f/f*^:*Mesp1Cre* mutants, the phosphor-Erk signal was also abolished in mesenchymal cells derived from neural crest in the distal OFT and first branchial arches. A higher number of apoptotic cells in the first branchial arches were detected (S3E and S3F Fig).HS is an upstream regulator of FGF signaling, but our data suggest that exogenous FGF can partially rescue the EMT and cell adhesion phenotypes. Since cells migrating from OFT explants originate from mesodermal precursors (S4A Fig), it is likely that *Ext1* deletion does not completely block FGF signaling in the mesoderm so that pathway is still stimulated by high doses of exogenous FGF ligand. On the other hand, pharyngeal explants contain a mixed cell population derived from the mesoderm and neural crest cells. Following access to exogenous FGF8b, neural crest cells are activated, partly compensating for mesodermal defects. Our data further confirm that *Ext1* expression in the SHF is required to maintain FGF signaling in these cells. Although molecular cross-talk between the SHF and neural crest has been reported, our experiments showed that loss of *Ext1* in neural crest cells does not affect FGF signaling in the SHF, but reduces FGF downstream genes in the OFT cushion. Accordingly, we propose that *Ext1* functions as a *ci*-*s*regulator of FGF signaling in the SHF or neural crest cells during OFT formation.

Previous studies have reported that deletion of FGF receptors in neural crest cells does not alter OFT morphogenesis, while reduction of FGF8 affects neural crest cell survival in the pharyngeal region [45–47]. Furthermore, proper spatial activation of Erk in the neural crest is important for cardiac conotruncal development. Although our results showed decreased expression of FGF downstream genes in the OFT cushion, downregulation of FGF signaling may not directly contribute to cardiovascular defects in *Ext1* ^*f/f*^:*Wnt1Cre* mutants. Pharyngeal arch arteries are the transient structures in cardiovascular development. A number of great artery anomalies originate from abnormal regression of arches. In our experiments, one of the sixth arch arteries disappeared earlier in *Ext1* ^*f/f*^:*Wnt1Cre* mice. We additionally detected a reduction of neural crest-derived cells in the OFT in mutant embryos (Fig 8Q). These data indicate that the molecular basis of OFT defects in *Ext1* ^*f/f*^:*Wnt1Cre* mutants is different from that in *Ext1* ^*f/f*^:*Mef2cCre* mutants, with distinct pathways potentially responsible for the cardiac phenotype.

*Tbx1* and *Six1/Eya* directly regulate *FGF8* expression [28]. In our study, both genes remained unaffected in the mutants, in contrast to the downstream gene, *Isl1* (Fig 4). *Ext1* may thus function independently of *Tbx1/Six1* to regulate the FGF8 signaling pathway. Notably, expression levels of *Wnt11* and *Bmp4* were downregulated upon *Ext1* deletion (Fig 3M–3P). Since HS can regulate various signaling ligands [5], partial rescue of the OFT defects by exogenous FGF8 suggests that *Ext1* is implicated in additional signaling pathways in OFT development. Nevertheless, data from the current study have provided strong evidence that *Ext1* plays an essential role in OFT formation by affecting mesodermal and neural crest cells. Further clarification of the functions of HS in cardiac progenitor cells is necessary for understanding the pathogenesis of congenital cardiac diseases related to GAGs.

## Supporting Information

S1 Fig Genes in the endocardium and myocardium are not affectedin*Ext1* ^*f/f*^:*Mesp1Cre*mice. (A,B) The master gene for cardiogensis, *Nkx2*.*5*, was not affected upon deletion of *Ext1*. (C–H)*In situ* hybridization analysis was performed to analyze expression of genes required for myocardium, *hand1*(C,D), *mlc2a* (E,F) and *Myc* (G,H). (I–L) The endocardial markers, *neuregulin* (I, J) and CD31 (K,L arrowhead), were detected via *in situ* hybridization and immunostaining, respectively. Levels of these genes were not changed in the mutant embryos. All embryos were examined at the 16- to 18-somite stage. V: ventricle. Scale bar: A–F, I–L: 50μm, G,H: 100μm.
(TIF)

S2 Fig SHF is affected in the *Ext1* ^*f/f*^:*Mesp1Cre*mutant. (A) Quantitative data on SHF cells in the boxed region in Fig 4C–4F (n = 3).(B) Quantification of cells in the splanchnic mesoderm at the 8- to 10-somite stage. Three embryos were used for each allele, and at least three sections counted for each embryo. (C,D) *In situ* hybridization revealed that *Isl1*is reduced in the splanchnic mesoderm in *Ext1* ^*f/f*^:*Mesp1Cre* embryos at the 16- to 18-somite stage(D). (E,F) As a positive control,*Isl1*was equally expressed in the ventral spinal cord in control and *Ext1* ^*f/f*^:*Mesp1Cre* embryos at the 16- to 18-somite stage. Scale bar: 50μm.
(TIF)

S3 Fig Deletion of *Ext1* downregulates FGF downstream genes, but not *FGF8*, in *Ext1* ^*f/f*^:*Mesp1Cre* embryos. (A–D) Deletion of *Ext1* did not alter the *FGF8* mRNA level in the splanchnic mesoderm in *Ext1* ^*f/f*^:*Mesp1Cre* embryos at the 10-somite stage. (E,F) Higher levels of apoptotic cells were detected in the first branchial arch in*Ext1* ^*f/f*^:*Mesp1Cre* embryos at the 13- to 14-somite stage (arrows).Scale bar: 50μm.
(TIF)

S4 Fig Cell lineage assay on the migrated cells of explants obtained by crossing *Mesp1Cre* with *ROSAmT/mG* transgenic mice. (A)Cells labeled with GFP migrated from OFT explants and underwent EMT (arrow). (B) Cells growing out from the pharyngeal explants include GFP-positive(arrow) and-negative cells (arrowhead). Cells derived from the mesoderm are labeled with GFP (green). Scale bar: 50μm.
(TIF)
