# Supplementary figures and images for: Heparan Sulfate Biosynthesis Enzyme, Ext1, Contributes to Outflow Tract Development of Mouse Heart via Modulation of FGF Signaling
Source: PLoS One. 2015 Aug 21;10(8):e0136518. doi: 10.1371/journal.pone.0136518 (PMC4546591; doi:10.1371/journal.pone.0136518)

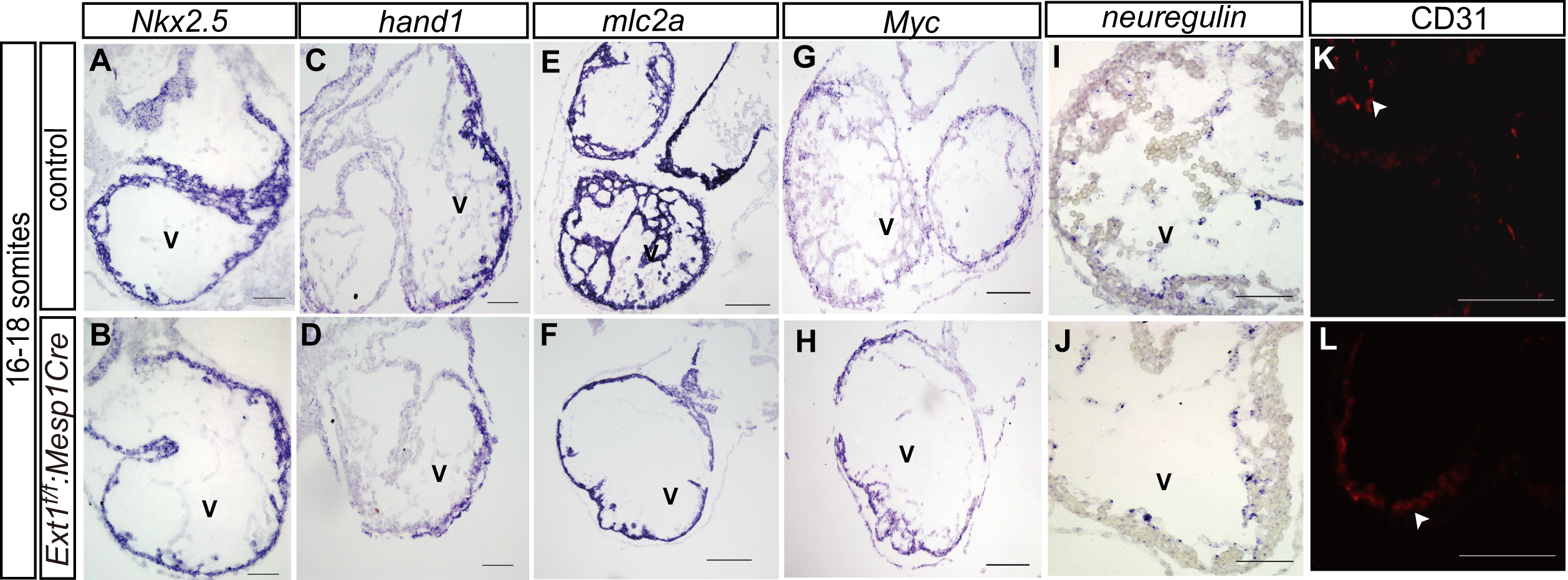

Supplement: S1 Fig — (A,B) The master gene for cardiogensis, Nkx2.5, was not affected upon deletion of Ext1. (C–H)In situ hybridization analysis was performed to analyze expression of genes required for myocardium, hand1(C,D), mlc2a (E,F) and Myc (G,H). (I–L) The endocardial markers, neuregulin (I, J) and CD31 (K,L arrowhead), were detected via in situ hybridization and immunostaining, respectively. Levels of these genes were not changed in the mutant embryos. All embryos were examined at the 16- to 18-somite stage. V: ventricle. Scale bar: A–F, I–L: 50μm, G,H: 100μm. (TIF) [file pone.0136518.s001.tif]

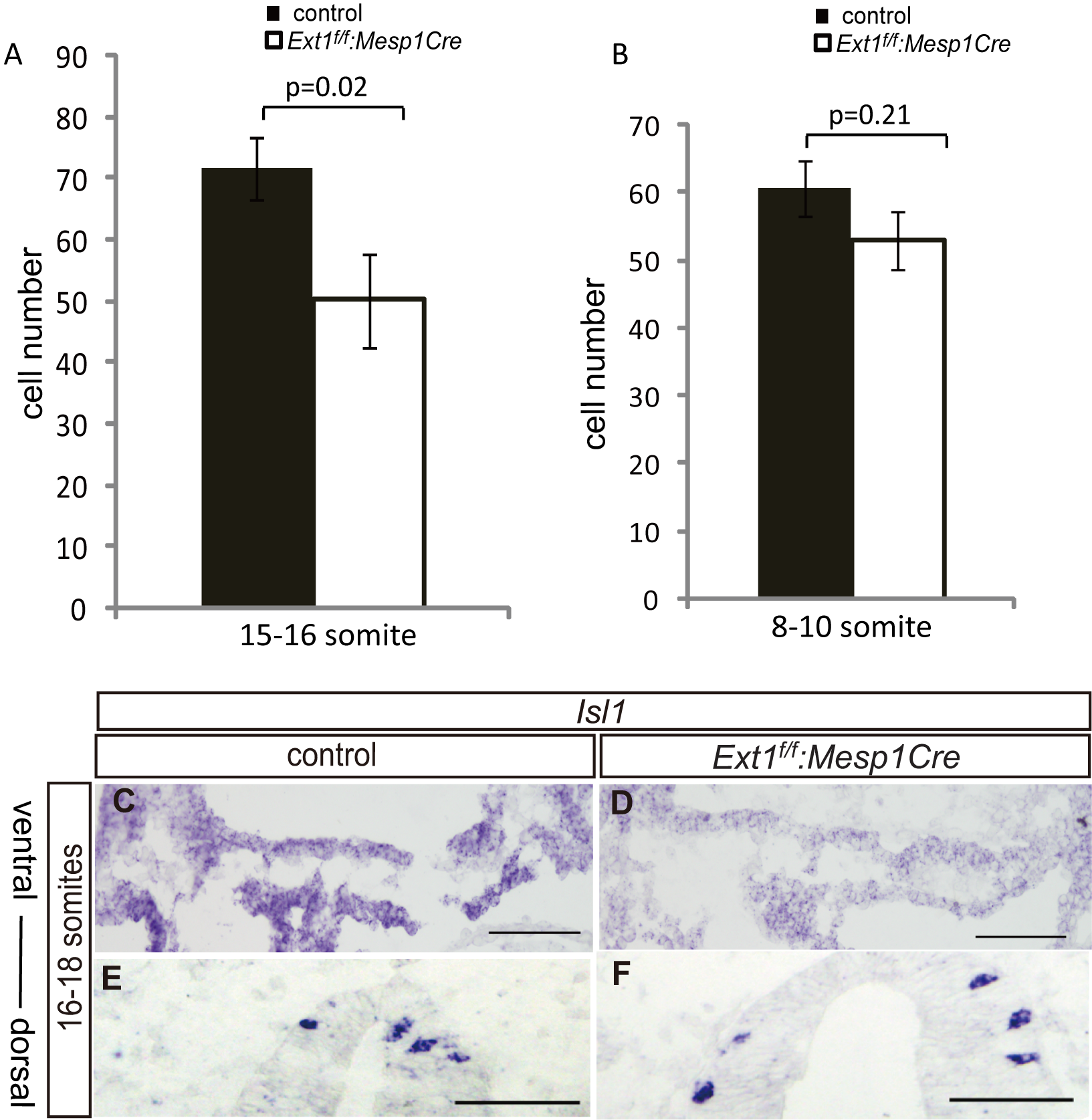

Supplement: S2 Fig — (A) Quantitative data on SHF cells in the boxed region in Fig 4C–4F (n = 3).(B) Quantification of cells in the splanchnic mesoderm at the 8- to 10-somite stage. Three embryos were used for each allele, and at least three sections counted for each embryo. (C,D) In situ hybridization revealed that Isl1is reduced in the splanchnic mesoderm in Ext1 f/f:Mesp1Cre embryos at the 16- to 18-somite stage(D). (E,F) As a positive control,Isl1was equally expressed in the ventral spinal cord in control and Ext1 f/f:Mesp1Cre embryos at the 16- to 18-somite stage. Scale bar: 50μm. (TIF) [file pone.0136518.s002.tif]

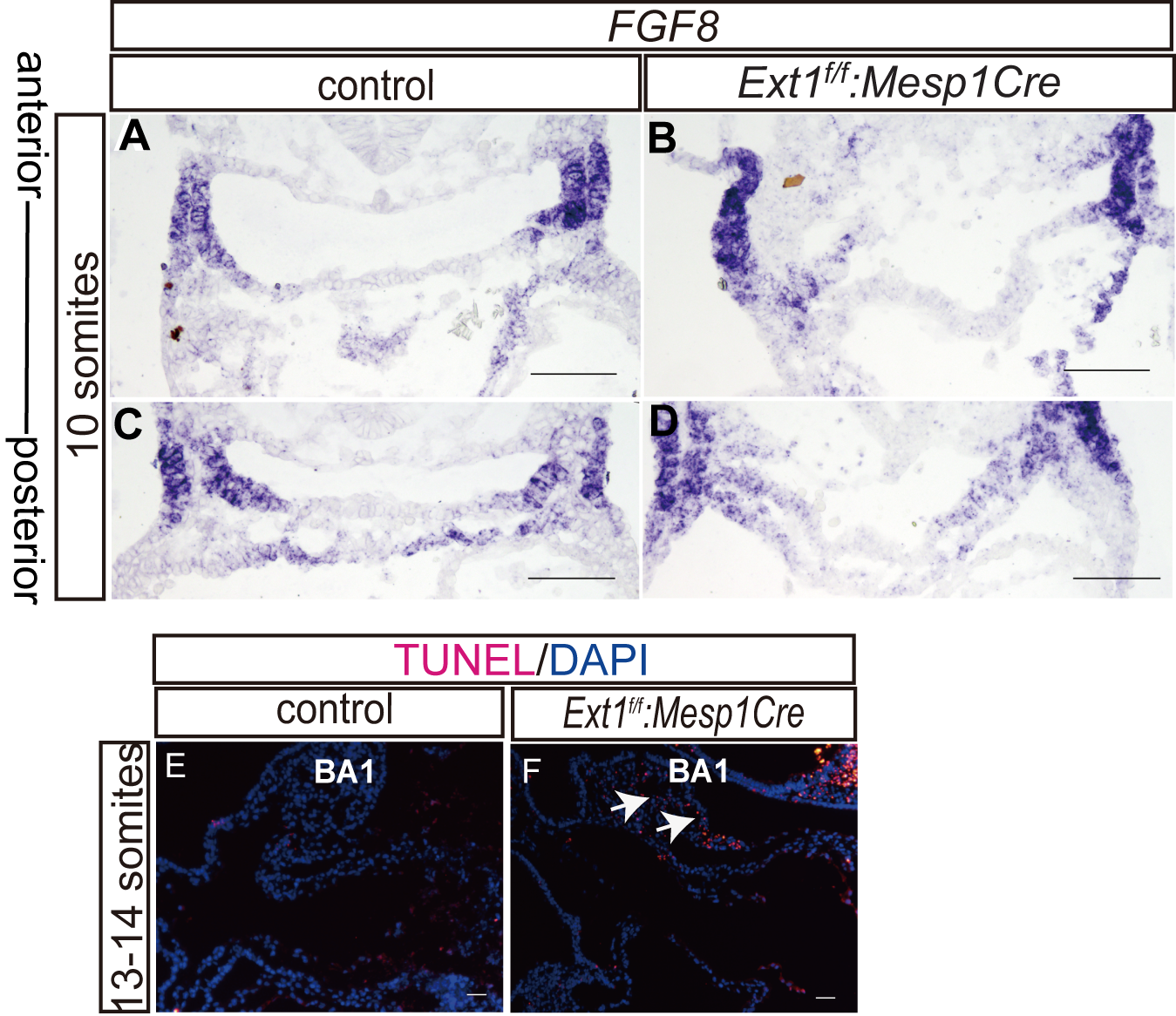

Supplement: S3 Fig — (A–D) Deletion of Ext1 did not alter the FGF8 mRNA level in the splanchnic mesoderm in Ext1 f/f:Mesp1Cre embryos at the 10-somite stage. (E,F) Higher levels of apoptotic cells were detected in the first branchial arch inExt1 f/f:Mesp1Cre embryos at the 13- to 14-somite stage (arrows).Scale bar: 50μm. (TIF) [file pone.0136518.s003.tif]

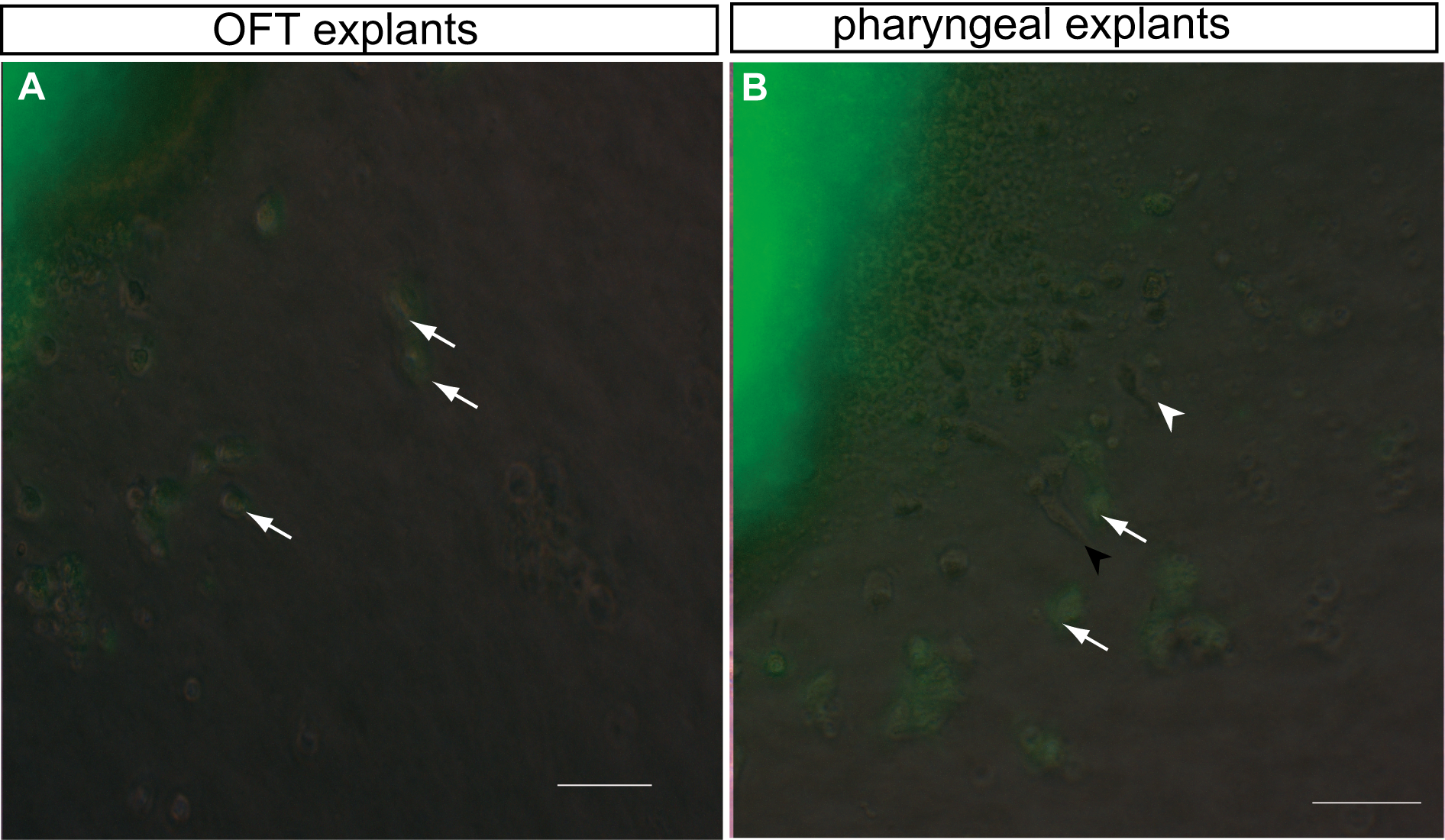

Supplement: S4 Fig — (A)Cells labeled with GFP migrated from OFT explants and underwent EMT (arrow). (B) Cells growing out from the pharyngeal explants include GFP-positive(arrow) and-negative cells (arrowhead). Cells derived from the mesoderm are labeled with GFP (green). Scale bar: 50μm. (TIF) [file pone.0136518.s004.tif]
